# 1004. Dalbavancin Use and Associated Cost-Savings: An Update

**DOI:** 10.1093/ofid/ofac492.845

**Published:** 2022-12-15

**Authors:** Amber C Streifel, Monica K Sikka, James S Lewis

**Affiliations:** Oregon Health and Science University, Portland, Oregon; Oregon Health and Science University, Portland, Oregon; Oregon Health and Science University, Portland, Oregon

## Abstract

**Background:**

Dalbavancin provides an alternative to standard of care intravenous (IV) antibiotics for the treatment of complicated infections, although clinical data continues to evolve In the United States, the use of dalbavancin as an alternative for patients requiring long courses of IV therapy can result in reduction of hospital days, and thus significant cost-savings to health systems.

**Methods:**

We conducted a retrospective review of dalbavancin use at our institution and resulting cost avoidance from 2015 to 2021. We identified all patients age 18 years or older who received > one dose of dalbavancin via medication records. Cost calculation was based on the number of outpatient dalbavancin days as a measure of avoided inpatient days.

**Results:**

189 patients received at least one dose of dalbavancin over the study period. The mean age 47.6 years and the most common infections were non-vertebral bone and joint infections (85; 45%), vertebral osteomyelitis (28; 14.8%) and skin and soft tissue infections (27; 14.3%). The most commonly isolated organism was *Staphylococcus aureus (109; 57.6%)*, MRSA (60; 31.7%) and MSSA (49; 25.9%). Dalbavancin dosing regimens varied over the study period. Reasons for selection of dalbavancin included history of injection drug use (67; 35.4%), lack of a safe home environment to receive daily IV antibiotics (26; 13.8%), and patient declining PICC or daily outpatient IV antibiotics (26; 13.8%). 84 patients (44%) received at least one dose of dalbavancin in the inpatient setting. Adverse effects were documented for 21 (11%) of patients. Readmission within 30 days of dalbavancin dose occurred for 27 (14.2%) patients, 14 (7.4%) of which were related to either infection or dalbavancin adverse effect. Relapse or recurrence of infection at 30 days was documented for 9 patients (4.8%). A total of 4,273 out-of-hospital dalbavancin days were identified, resulting in a cost savings of $10,464,382 or a mean of $55,367.10 per patient.

**Figure:**
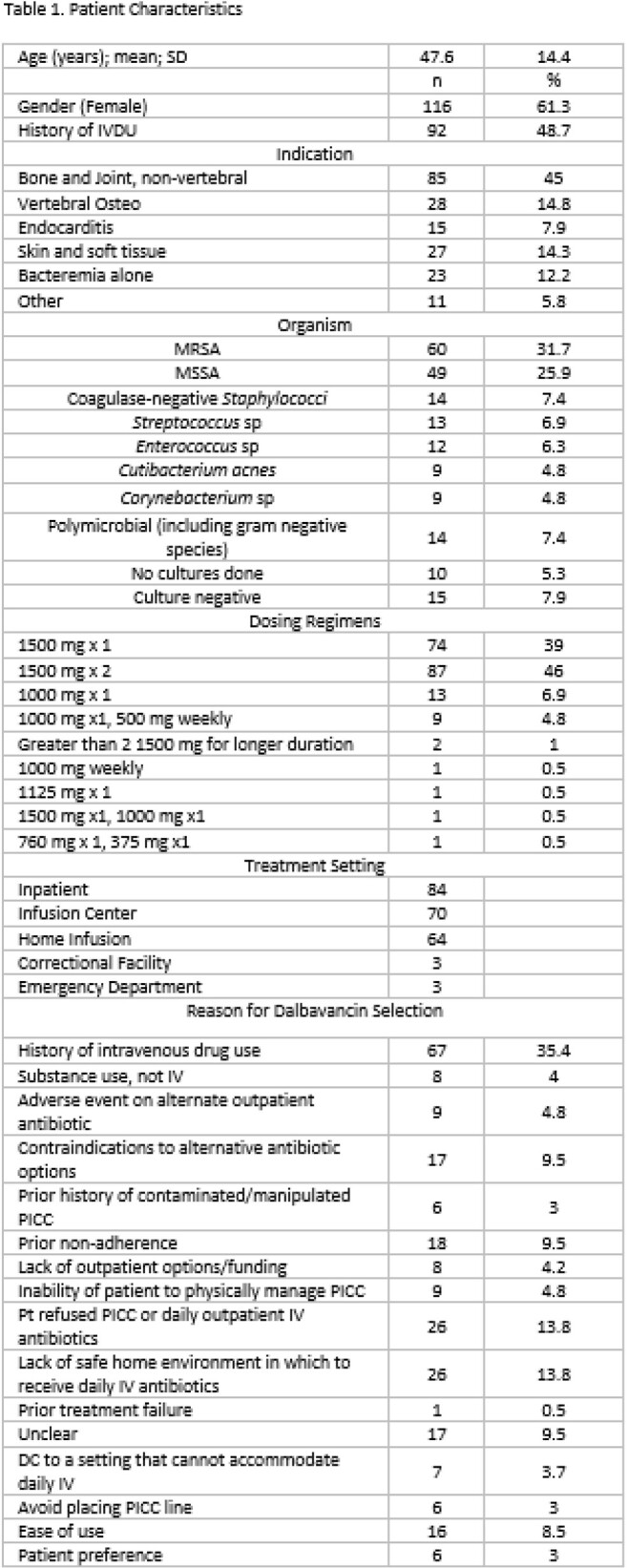

**Figure:**
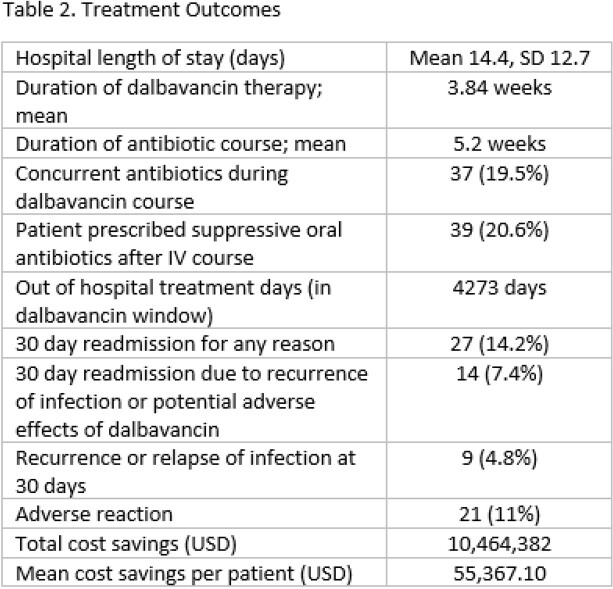

**Conclusion:**

As the use of dalbavancin for complicated infections continues to increase, the potential for health system cost-savings increases, driven by reduction in length of hospital stay. Prospective cost-analysis research is needed to further describe the cost benefits associated with dalbavancin use to patients and health systems in the United States.

**Disclosures:**

**Monica K. Sikka, MD**, F2G: Site research investigator **James S. Lewis, PharmD, FIDSA**, Cidara: Advisor/Consultant|Merck: Advisor/Consultant|SeLux Diagnostics: Advisor/Consultant.

